# Promotion of diced cartilage survival and regeneration with grafting of small intestinal submucosa loaded with urine‐derived stem cells

**DOI:** 10.1111/cpr.13542

**Published:** 2023-09-18

**Authors:** Shang Li, Rui Wang, Liping Huang, Yanlin Jiang, Fei Xing, Weiqiang Duan, Ying Cen, Zhenyu Zhang, Huiqi Xie

**Affiliations:** ^1^ Department of Orthopedic Surgery and Orthopedic Research Institute, Laboratory of Stem Cell and Tissue Engineering, State Key Laboratory of Biotherapy, West China Hospital Sichuan University Chengdu Sichuan China; ^2^ Department of Plastic and Burn Surgery, West China Hospital Sichuan University Chengdu Sichuan China; ^3^ Medical Cosmetic Center, Beijing Friendship Hospital Capital Medical University Beijing China; ^4^ Department of Plastic Reconstructive and Aesthetic Surgery, West China Tianfu Hospital Sichuan University Chengdu Sichuan China; ^5^ Frontier Medical Center Tianfu Jincheng Laboratory Chengdu Sichuan China

## Abstract

Cartilage absorption and calcification are prone to occur after the implantation of diced cartilage wrapped with autologous materials, as well as prolong the operation time, aggravate surgical trauma and postoperative pain during the acquisition process. Small intestinal submucosa (SIS) has suitable toughness and excellent degradability, which has been widely used in the clinic. Urine‐derived stem cells (USCs), as a new type of stem cells, have multi‐directional differentiation potential. In this study, we attempt to create the tissue engineering membrane material, termed USCs‐SIS (U‐SIS), and wrap the diced cartilage with it, assuming that they can promote the survival and regeneration of cartilage. In this study, after co‐culture with the SIS and U‐SIS, the proliferation, migration and chondrogenesis ability of the auricular‐derived chondrocyte cells (ACs) were significantly improved. Further, the expression levels of chondrocyte phenotype‐related genes were up‐regulated, whilst that of dedifferentiated genes was down‐regulated. The signal pathway proteins (*Wnt3a* and *Wnt5a*) were also participated in regulation of chondrogenesis. In vivo, compared with perichondrium, the diced cartilage wrapped with the SIS and U‐SIS attained higher survival rate, less calcification and absorption in both short and long terms. Particularly, USCs promoted chondrogenesis and modulated local immune responses via paracrine pathways. In conclusion, SIS have the potential to be a new choice of membrane material for diced cartilage graft. U‐SIS can enhance survival and regeneration of diced cartilage as a bioactive membrane material.

## Graphical abstract

Schematic illustration of the study design. In vitro, the effects of SIS and U‐SIS on the behaviour of chondrocyte: cell proliferation, migration, chondrogenic ability, regulation of protein secretion and changes in genes and signal pathway protein related to cartilage phenotype. In vivo, perichondrium, SIS and U‐SIS wrapped diced cartilage were used for grafting and their effect on cartilage repair was evaluated in a rabbit ear cartilage defect model.

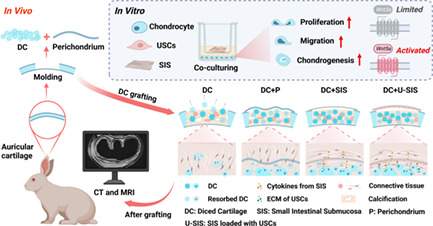

## 1 INTRODUCTION

Cartilage is a widely used autologous tissue in rhinoplasty for its advantages such as biocompatibility, low risk of infection, cost‐effectiveness and ample graft volume compared with alloplastic implants.^1^ As we all know, cartilage graft applied in plastic surgery is to reinstate the volume, structural unity and strength in the corresponding parts of the body.^2^ During the past decade, the technique of grafted cartilage has been improved constantly,^2^ with examples including concentric carved costal cartilage,^3^ oblique splitting cartilage,^4^, ^5^ transverse slicing cartilage,^6^ accordion carved cartilage^7^ and diced cartilage graft.^8^ The primary purpose of such techniques is to decrease the rate of cartilage warping after cartilage graft, which may seriously affect the postoperative appearance due to changes of mechanical property of the inner cartilage and its microenvironment.^9^ However, clinical studies have suggested that all solid grafts are associated with cartilage warping, which can cause failure of the operation.^10^, ^11^ As a result, diced cartilage graft has been proposed to prevent cartilage warping by slicing it into dices and disrupting its inner interactions.^12^

So far diced cartilage graft has been widely used in rhinoplasty repairing various forms of nasal deformities. Comparing with other solid cartilage graft, it requires less volume of cartilage, is flexible in shape and size, and has less graft contour visibility,^8^ which can reduce the damage at the donor site and requirement of surgical skills. As shown by many clinical trials, free diced cartilage without any wrapping materials has the potential to be palpable, visible, absorbed and uncontrolled dispersion after the grafting, resulting in post‐operative irregularities.^13^ Therefore, it is necessary to use wrapping materials to cover and shape the transplanted diced cartilage. During clinical treatment, perichondrium,^14^ muscle fascia^15^, ^16^ and AlloDerm® (LifeCell Corporation, Branchburg, NJ)^17^ are commonly used as wrapping materials, whilst each has certain limitations during the operation. The muscle fascia, including deep temporal fascia, rectus abdominal fascia and pectoralis major fascia, is a favourable wrapping material for its abundance in the body,^18^ but it usually has distinct harvesting site for the donor cartilage with longer operative time, larger incision, and severe perioperative pain.^15^, ^19^, ^20^ The AlloDerm® has a high cost and is associated with cartilage resorption.^21^ The perichondrium, as another favourable wrapping material, could improve the viability and survival rate of the graft cartilage^22^ and improve its biomechanical properties,^23^ though limited source has restricted its usage in the operations. Therefore, none of such materials was considered as ideal for wrapping the diced cartilage. To identify a wrapping material with the potential to substitute autologous tissue with richness in resources and abilities of improving diced cartilage viability and survival rate is urgent.

Small intestinal submucosa (SIS), as a natural decellularised biomaterial consisting of collagen and various growth factors, could promote tissue regeneration, angiogenesis and immunoregulation.^24^, ^25^ In recent years, the SIS has been widely used in tissue regeneration engineering and clinical trials for bladder repairing,^26^ gastric ulcer repairing,^27^ inguinal hernia repairing^28^ and breast reconstruction.^29^ These studies have confirmed that the SIS possessed great biocompatibility and safety, as well as ability to promote tissue regeneration in vivo, which is essential for diced cartilage grafting. Moreover, the SIS has also been used for cartilage‐related repair and regeneration such as tracheal cartilage regeneration,^30^, ^31^ meniscal cartilage repairing,^32^, ^33^, ^34^ upper airway regeneration^35^ and rheumatoid arthritis repairing.^36^ The results proved that the SIS could promote the secretion of extracellular matrix (ECM) by chondrocytes during in vitro chondrogenesis and in vivo cartilage regeneration.^30^, ^37^ More importantly, the SIS has suitable toughness and excellent degradability. For diced cartilage grafting, it is crucial that the wrapping material possesses flexibility and ductility. Therefore, in this study, we have chosen the SIS as the wrapping material and explored its feasibility for the shaping of the transplanted diced cartilage during auricular cartilage tissue engineering.

Urine‐derived stem cells (USCs) is a type of mesenchymal stem cells (MSCs).^38^ Comparing with MSCs derived from other sources, the USCs have the advantages of simple and non‐invasive acquisition,^39^, ^40^ rapid proliferation^41^ and multidirectional differentiation potential. As a result, the USCs isolated from autologous urine have been applied in various cell treatment. Previous studies have also shown that the ECM of the USCs has chondrogenic capacity.^42^, ^43^

**FIGURE 1:**
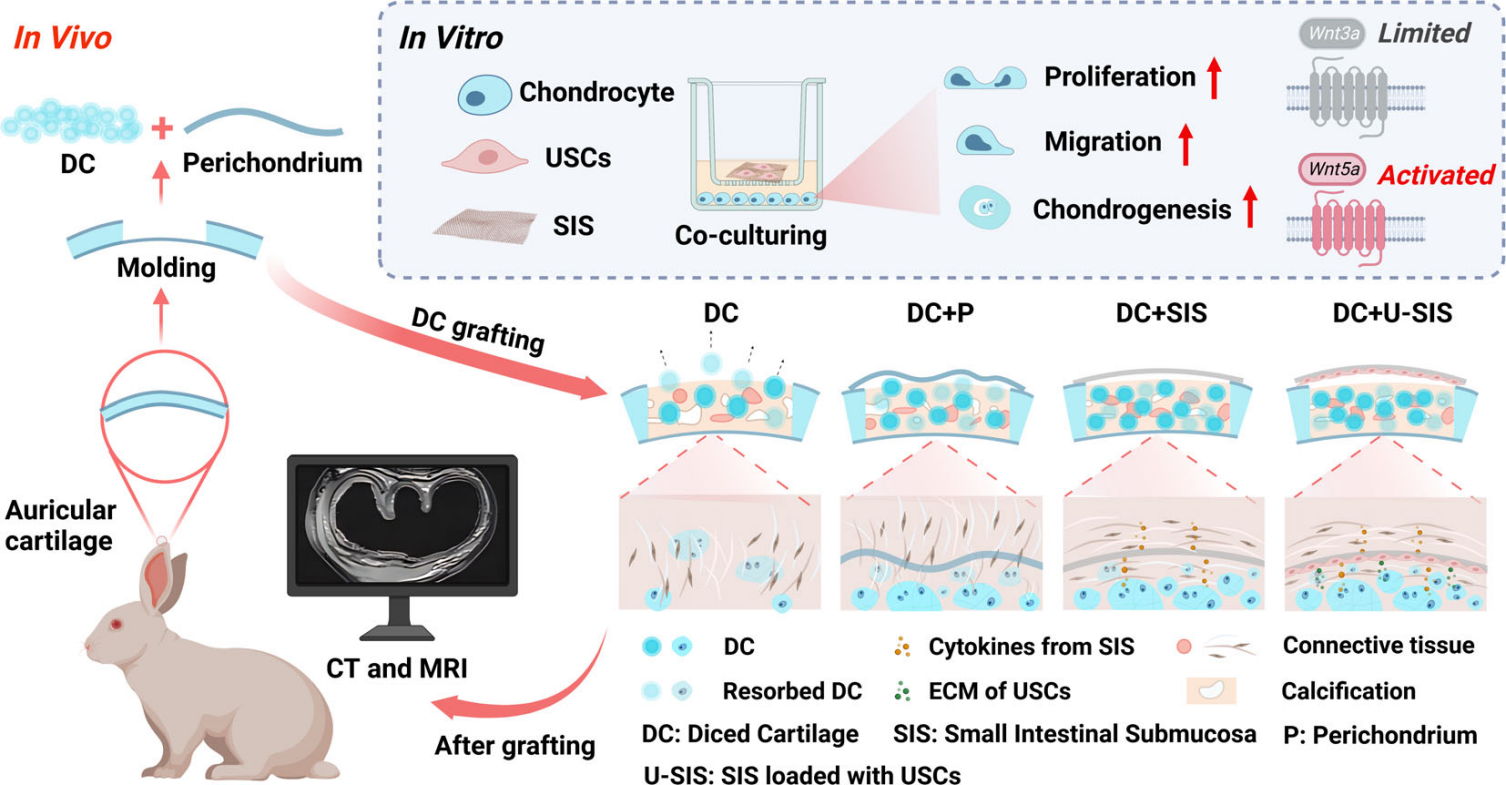
Schematic illustration of the study design. In vitro, the effects of small intestinal submucosa (SIS) and U‐SIS on the behaviour of chondrocyte: cell proliferation, migration, chondrogenic ability, regulation of protein secretion and changes in genes and signal pathway protein related to cartilage phenotype. In vivo, perichondrium, SIS and U‐SIS wrapped diced cartilage were used for grafting and their effect on cartilage repair was evaluated in a rabbit ear cartilage defect model.

Based on above discoveries, we have cultured the USCs on the surface of the SIS to derive a U‐SIS membrane with enhanced chondrogenic capacity. Both the SIS and U‐SIS membrane were used for wrapping the diced cartilage and grafted into cartilage defects in rabbit models, to evaluate their ability to enhance the survival of diced cartilage and the underlying mechanism (Figure 1).

## 2 MATERIALS AND METHODS

### 2.1 Primary culture of the USCs

Primary culture of the USCs was carried out with a previously described method.^44^ The USCs were obtained from fresh amicrobic urine from young male donors (20 ~ 30 years old). The research protocols were approved by the Ethics Committee on Biomedical Research, West China Hospital of Sichuan University (No. 20211066). The urine sample (about 200 mL) was washed with phosphate‐buffered saline (PBS; ZSGB‐BIO, China) and centrifuged for twice. The sediment was re‐suspended and cultured in T25 cell culture flasks (Corning, USA) containing 5 mL of USCs medium.^44^ The medium was replaced 7 days after the cell seeding, with removal of non‐adherent cells.

### 2.2 Primary culture of the rabbit auricular‐derived chondrocyte cells (ACs)

Auricular cartilage was separated from New Zealand rabbits' ears without perichondrium and cut into pieces after washing with iodophor and PBS. The cartilage pieces were digested with trypsin on a 37°C shaker and 0.25% collagenase (Type II, Vetec™, Sigma‐Aldrich, USA) thereafter. The sediment was then re‐suspended and cultured in T25 cell culture flasks (Corning, USA) containing 5 mL of culture medium comprised 90% Dulbecco's modified Eagle medium (DMEM, Gibco, USA) and 10% foetal bovine serum (FBS, Gibco, USA) supplemented by 100 units/mL penicillin (Gibco, USA) and 100 μg/mL streptomycin (Gibco, USA). The medium was replaced 4 days after the seeding with removal of non‐adherent cells.

### 2.3 Preparation and characterisation of the SIS and U‐SIS

#### 2.3.1 Preparation of the SIS and U‐SIS

The SIS was prepared with a previously described method.^24^ The muscularis, mucosa and serosa layers of porcine intestine were cleaned by shaving. The residual membranes were soaked in chloroform–methanol solution (1:1, V:V) to remove the adipose tissue. After washing with water, the membranes were soaked in trypsin and SDS in sequence for decellularisation. Thereafter, the membranes were washed with pure water and lyophilized.

To prepare the U‐SIS, the SIS was cut into pieces with a size of 2.0 × 1.5 cm and sterilised with epoxyethane at 37°C. The SIS pieces were soaked in USCs medium for 1 h. Thereafter, 2.0 × 10^4^ USCs were seeded onto each piece of SIS and cultured for 7 days.

#### 2.3.2 Scanning electron microscopy (SEM)

The lyophilized SIS were directly sputtered with gold and observed under SEM (JEOL, Japan).

The U‐SIS samples were soaked in 10% formaldehyde for 30 min at 4°C, dehydrated through graded ethanol, and went through critical‐point‐drying, sputtered with gold and subjected to SEM.

#### 2.3.3 Live/dead staining

The USCs and ACs were respectively seeded onto the surface of the SIS and cell culture flasks. The samples were soaked with 1 μM calcein AM (Sigma, USA) and 1 μM propidium iodide (Roche, USA) for 30 min in the darkness, washed three times with PBS, and visualised under a confocal microscope (A1RMP+, Nikon, Japan).

### 2.4 Co‐culturing of the ACs with the SIS and U‐SIS

In the 12‐well cell culture plates (Coring, USA), 2.0 × 10^4^ ACs were seeded into each well containing 1.5 mL of cell culture medium. After the cell adherence, the SIS and U‐SIS were put in the transwell chambers, one membrane for each chamber containing 1.5 mL cell culture medium. The medium was replaced another day. The cells were divided into three groups: (1) ACs group, where the ACs were cultured in standard culture medium; (2) ACs + SIS group, where the ACs was co‐cultured with the SIS in standard culture medium; (3) ACs + U‐SIS group, where the ACs was co‐cultured with the U‐SIS in standard culture medium.

#### 2.4.1 Proliferation of the co‐cultured ACs

After the start of co‐culturing, the proliferation of the ACs was recorded daily with a Cell Counting Kit‐8 (CCK‐8) (Dojindo Molecular Technologies, Japan). The absorbance was measured at 450 nm (OD_450_) with a spectrophotometer (Thermo, USA).

#### 2.4.2 Migration of the co‐cultured ACs

The migration of ACs was recorded with a cell scratch assay. When the co‐cultured ACs reached 95% confluence, a pipette was used to scratch the cells in the middle of wells. The cell culture medium was replaced by a low serum concentration which comprised 95% DMEM (Gibco, USA) and 5% FBS (Gibco, USA) supplemented by 100 units/mL penicillin (Gibco, USA) and 100 μg/mL streptomycin (Gibco, USA). Cell‐covered area was recorded under a microscope at 0, 6, 12, 18, and 24 h, and the migration rate was calculated as [1 − (pixel size of uncovered area at each time point)/(pixel size of initially scratched area)] × 100%.^45^

#### 2.4.3 Glycosaminoglycan (GAGs) assay of the co‐cultured ACs

Sulphated GAG Assay (Blyscan, UK) was used for the measurement. The co‐cultured ACs were tested on days 4, 7 and 11. The ACs were washed with PBS and soaked in 1,9‐dimethylmethylene blue staining for 1 h at 37°C. The residual staining was removed thereafter; 1.5 mL dissociation reagent was added into each well to release the dye. Subsequently, the absorbance of solution was measured at 656 nm (OD_656_) with a spectrophotometer to determine the relative GAGs content.

#### 2.4.4 Preparation of conditional medium of the SIS and U‐SIS

The SIS was soaked in cell culture medium for 24 h at 37°C. Thereafter, the SIS was removed, and the residual liquid (conditional medium) was collected. The ratio between the SIS and medium was calculated as 6 × Volume of medium = 2 × Square of SIS.

To obtain the conditional medium of the U‐SIS, 4.0 × 10^4^ USCs were seeded onto the surface of the SIS with a size of 1.0 × 1.5 cm. The U‐SIS was cultured in 12‐well cell culture plates until the adherence of the cells. Thereafter, the USCs medium was removed, and 2 mL cell culture medium was added to each well. The U‐SIS was cultured at 37°C, 5% CO_2_ for 24 h, and the medium (conditional medium of the U‐SIS) was collected into a tube.

### 2.5 Pellet culture of the ACs

The ACs was cultured in a 3D environment for pellet culture with the leach liquor of the SIS and U‐SIS in the medium. 4.0 × 10^5^ ACs were added into a 15 mL centrifuge tube containing 1 mL cell culture medium. The tubes were centrifuged at 1200 rpm for 5 min with lid open, and cultured in 5% CO_2_ at 37°C, with vertical position. On days 3, the cells shrunk into a bowl shape and float in the medium. The pellets were divided into three groups: (1) ACs group, soaked in cell culture medium; (2) ACs + SIS group, soaked in cell culture medium plus leach liquor of the SIS; (3) ACs + U‐SIS group, soaked in cell culture medium plus the leach liquor of U‐SIS. The pellets were cultured for 14 days, with the medium replaced every other day.

### 2.6 Evaluation of gene transcript expression

For RT‐qPCR, co‐cultured ACs from days 4, 7 and 11 were used for the determination of the expression of *COL2*, *COL2/COL1*, aggrecan (*ACA*N) and *SOX9* genes. RNA was extracted with a Total RNA Extraction Kit (Eastep® Super, China). cDNA synthesis and PCR reaction was carried out with a PrimeScript RT reagent Kit gDNA Eraser with SYBR Green (Takara, USA) by following the instructions of the manufacturer. Expression of the genes was calculated by using the 2^−ΔCt^ and 2^−ΔΔCt^ methods.^46^ The values were calculated in relation to the *Ct* value of the *GAPDH* gene. The primers were showed in Table S1.

### 2.7 Western blotting

Western blotting was used to determine the expression of *Wnt3a* and *Wnt5a* proteins in the co‐cultured ACs. After the ACs were co‐cultured for 7 days, the medium was removed, and the cells were washed with PBS for three times. Thereafter, they were lysed with a RIPA lysis buffer (Epizyme, China) for 15 min at 0°C, and by ultrasound at 400 w for 40 s. The lysed cells were centrifuged at 4°C, 15,000× g for 15 min. The protein concentration of each sample was determined by using a BCA Protein assay kit (Epizyme, China) by following the instruction of the manufacturer. The extracted proteins were mixed with a loading buffer (Epizyme, China) and boiled at 100°C for 10 min. Equal amounts of protein were separated by 12.5% sodium dodecyl sulphate polyacrylamide gel electrophoresis and transferred to a polyvinylidene difluoride membrane (PVDF) (Hybond, USA). The PVDF membrane was blocked with 5% skim milk for 1 h at room temperature and probed with primary antibodies at 4°C overnight. The antibodies included Wnt3a (ABclonal; A0642; 1:1000), Wnt5a (ABclonal; A19133; 1:1000), β‐actin (ABclonal; AC026; 1:5000) and GAPDH (ABclonal; AC027; 1:5000). After washing with TBST, the membranes were incubated with secondary antibodies (ABclonal; AS014; 1:5000) at 37°C for 1 h. Chemiluminescent signals were generated with a chemiluminescence imaging kit (Thermo Fisher Scientific, USA). GAPDH was used as the internal control. The intensity of the bands was quantified using image processing and analysis in Java (ImageJ; National Institutes of Health) software.

### 2.8 Enzyme‐linked immunosorbent assays (ELISA)

ELISA was used to determine the concentration of type X collagen in the ACs. After the ACs were co‐cultured with the SIS and U‐SIS, total proteins were extracted as described in 2.7 on days 3, 7 and 11; 50 μL of samples and various concentrations of COL10 standard protein were added to each well on the plate, and 100 μL horseradish peroxidase‐labelled antibody was added to each well. The plate was incubated at 37°C for 60 min. After five rounds of washing, 100 μL working solution were added to each well to incubate at 37°C for 15 min. After the reaction, the absorbance of each well was determined at 450 nm with a spectrophotometer (Thermo, USA).

### 2.9 Animal experiments

All animal experiments were approved by the Animal Care and Use Committee of Sichuan University (No. 2020375A) and conformed to the Principles of Laboratory Animal Care formulated by the National Society for Medical Research.

#### 2.9.1 Preparation of diced cartilage graft

Twenty‐four male New Zealand rabbits (weighing 2.5 ± 0·5 kg each) were anaesthetised by pentobarbital sodium at a dose of 1.1 mL/kg. Two full‐thickness cartilage defects with a size of 0.5 × 1.0 cm were created at the back of each ear near the head, with an interval of 0.5 cm, so that four cartilage defects were created for each rabbit. The cartilage defects in each rabbit were divided into four groups: (1) the DC group, in which the cartilage defects were repaired by diced cartilage graft; (2) the DC + P group, in which the cartilage defects were repaired by diced cartilage graft with perichondrium wrapping; (3) the DC + SIS group, in which the cartilage defects were repaired by diced cartilage graft with SIS wrapping; (4) the DC + U‐SIS group, in which the cartilage defects were repaired by diced cartilage graft with U‐SIS wrapping (24 defects per group). The diced cartilage in each defect was made from the cartilage removed from the same site, which ensured the volume of the diced cartilage in each defect matched with that of the defect. The cartilages were cut into diced cartilage with a size of 0.5 ~ 1.0 mm, and the perichondrium of the cartilage was completely removed before dicing, and the skin was stitched.

Three male New Zealand rabbits (weighing 2.5 ± 0.5 kg each) were anaesthetised by pentobarbital sodium at a dose of 1.1 mL/kg. Four full‐thickness cartilage defects were created at each rabbit's ears as described above. The cartilage defects were used as the control group and received no treatment except skin stitching.

The animals were sacrificed by aeroembolism at 1, 2, 4, 8, 12 and 24 weeks.

#### 2.9.2 Histology and immunofluorescence analyses

The samples removed from the rabbits were soaked in 10% formaldehyde, dehydrated through gradient ethanol and embedded in paraffin. Subsequently, they were sectioned into 4 μm slices, followed by histological staining, including HE staining, Toluidine blue staining, Safranin O and Fast green staining, and Sirius red staining, to evaluated the survival and calcification of the cartilages.

HLA‐ABC antibody (BD Biosciences; 555,552; 1:1000) was used to observe the USCs in vivo. CD86 (ABclonal; A1199; 1:1000) and CD206 (Abcam; ab32575; 1:1000) antibodies were used to observe the local inflammatory reaction.

#### 2.9.3 Magnetic resonance imaging (MRI) and MicroCT scanning

The samples were scanned with a 3T nuclear magnetic resonance instrument, and the signals were collected by a GE 1.5T MRI scanner with 8‐way coils. A high‐resolution T1 sequence was used to obtain the transverse position scan. The parameters were set as: repetition time = 619 ms, echo time = 34 ms, flip angle = 19.23°, thickness = 1 mm, interval = 0, total layers = 21, filed of view = 6, image acquisition matrix = 288 × 244 and voxel size = 0.2 × 0.3 × 1 mm.

A GE eXplore Locus MicroCT scanner (General Company, USA) was used. The samples were placed at the same site of the shelf and scanned sequentially. The parameters were set as follows: scan voltage = 80 V, sweep current = 100 μA, total scanned layers = 512, scanning time = 14 min, resolution = 90 μm. Thereafter, a 360° continuous tomography was carried out for each sample, and a 50 × 50 × 50 mm^3^ area was selected for three‐dimensional reconstruction.

### 2.10 Statistical analysis

All data was presented as mean ± standard deviation. Statistical significance between the groups was determined by one‐way ANOVA and Student's *t*‐test. The *p* < 0.05 was statistically significant (*p < 0.05, **p < 0.01, ***p < 0.001).

## 3 RESULTS

### 3.1 Preparation and characterisation of the SIS and U‐SIS

**FIGURE 2:**
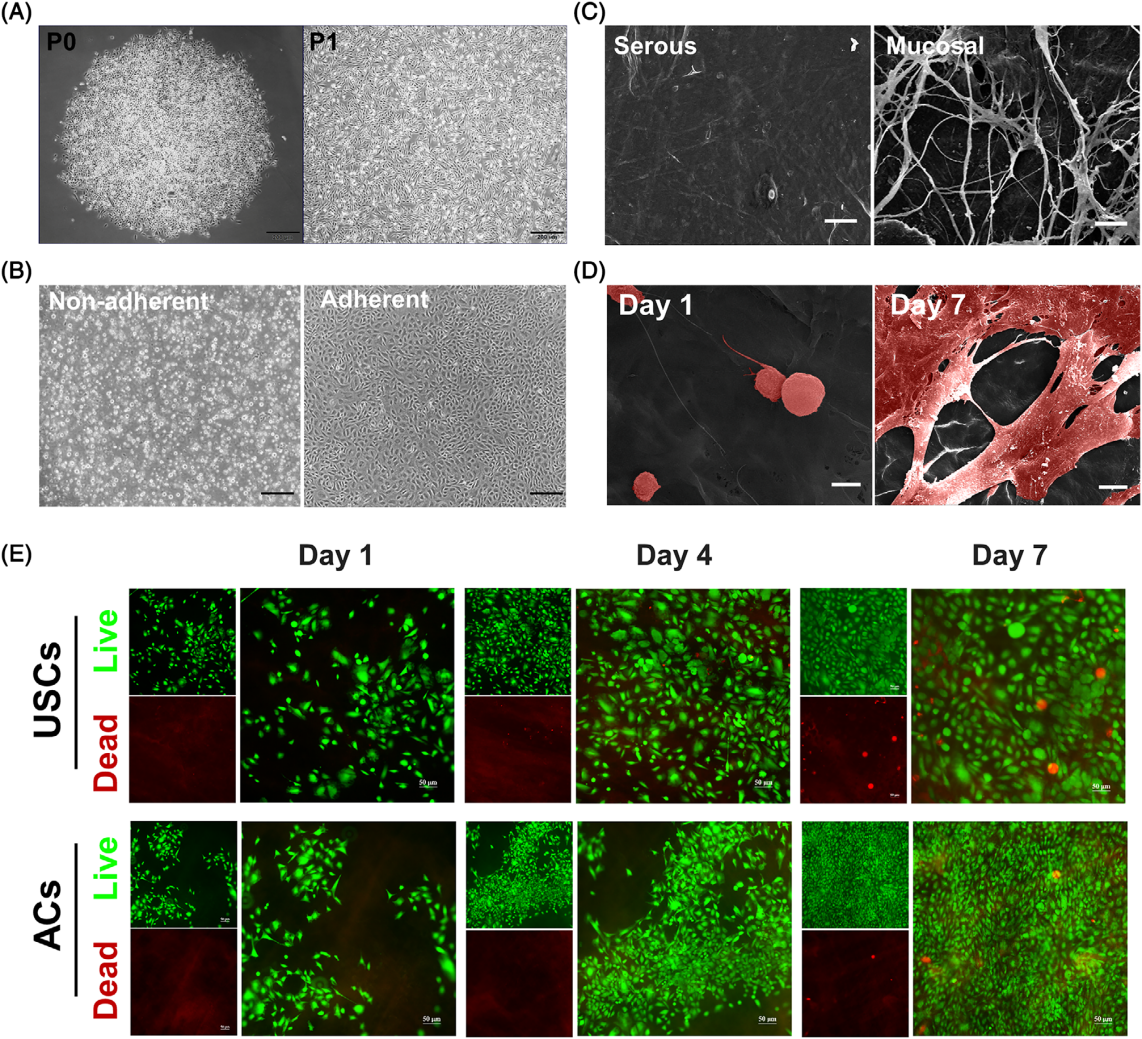
Characterisation of the urine‐derived stem cell (USCs), auricular‐derived chondrocyte cell (ACs), small intestinal submucosa (SIS) and U‐SIS. (A) Morphology of P0 and P1 USCs. Scale bar = 200 μm. (B) Morphology of non‐adherent and adherent ACs. Scale bar = 200 μm. (C) SEM image of mucosal and serous surfaces of the SIS. Scale bar = 50 μm. (D) Ultrastructure of the USCs seeded onto the surface of the SIS after 1 and 7 days of culture. Scale bar = 10 μm. (E) Live/dead staining of the USCs and ACs seeding onto the surface of the SIS after 1, 3 and 7 days of culture. Scale bar = 50 μm.

The USCs were extracted from urine,^47^ with the primary cell colonies formed within 7 days after the culture (Figure 2A). The ACs were isolated from rabbit's auricular cartilage (Figure 2B), and were polygon in shape after adherence.

The SIS was obtained after shaving and washing. To assess its cytocompatibility, live/dead staining was carried out after the cells were cultured on its surface after 1, 3 and 7 days (Figure 2E), and no obviously dead USCs or ACs were found. Following the culture, the cells spread well with a flattened morphology. By SEM, many interconnected pores were seen on its mucosal surface, whilst fewer pores were found on the serous surface (Figure 2C). After seeding, the USCs could be observed on the surface of the SIS. By day 7, the USCs have proliferated well and spread over the surface of the SIS, forming the U‐SIS membrane (Figure 2D).

#### 3.1.1 SIS and U‐SIS could promote cell proliferation and migration compared to ACs alone

**FIGURE 3:**
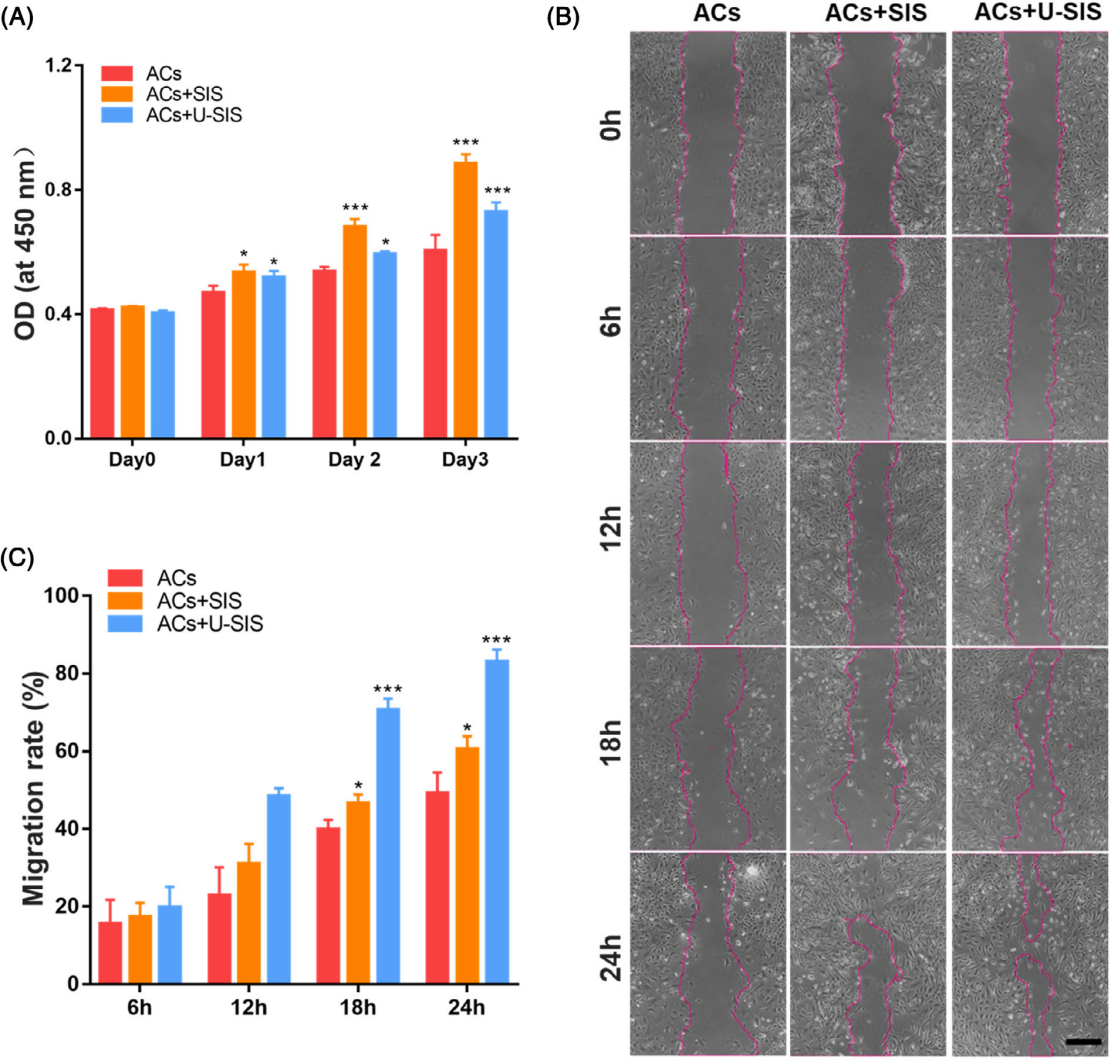
Effect of small intestinal submucosa (SIS) and U‐SIS on proliferation and migration of chondrocytes. Characterisation of the urine‐derived stem cell (USCs), auricular‐derived chondrocyte cell (ACs), SIS and U‐SIS. (A) Proliferation of the ACs co‐cultured with the SIS and U‐SIS measured by a CCK‐8 kit. *p < 0.05, ***p < 0.001. (B) Migration rate and (C) images of the ACs co‐cultured with the SIS and U‐SIS. *p < 0.05, ***p < 0.001.

As detected by the CCK‐8 assay (Figure 3A), the proliferation of the ACs co‐cultured with the SIS and U‐SIS were significantly faster compared with the ACs cultured alone. By the cell scratch assay (Figure 3B,C), the ACs have migrated to the blank area after scratching in 24 h. The cells in the ACs + U‐SIS (82.96% ± 3.21%) and AC + SIS groups (60.47% ± 3.36%) have migrated significantly faster compared with the ACs group (49.19% ± 5.35%). And those co‐cultured with the U‐SIS showed the greatest migration rate.

### 3.2 Chondrogenic ability of the ACs co‐cultured with the SIS and U‐SIS

#### 3.2.1 Expression level of GAGs and type II collagen in the ACs

**FIGURE 4:**
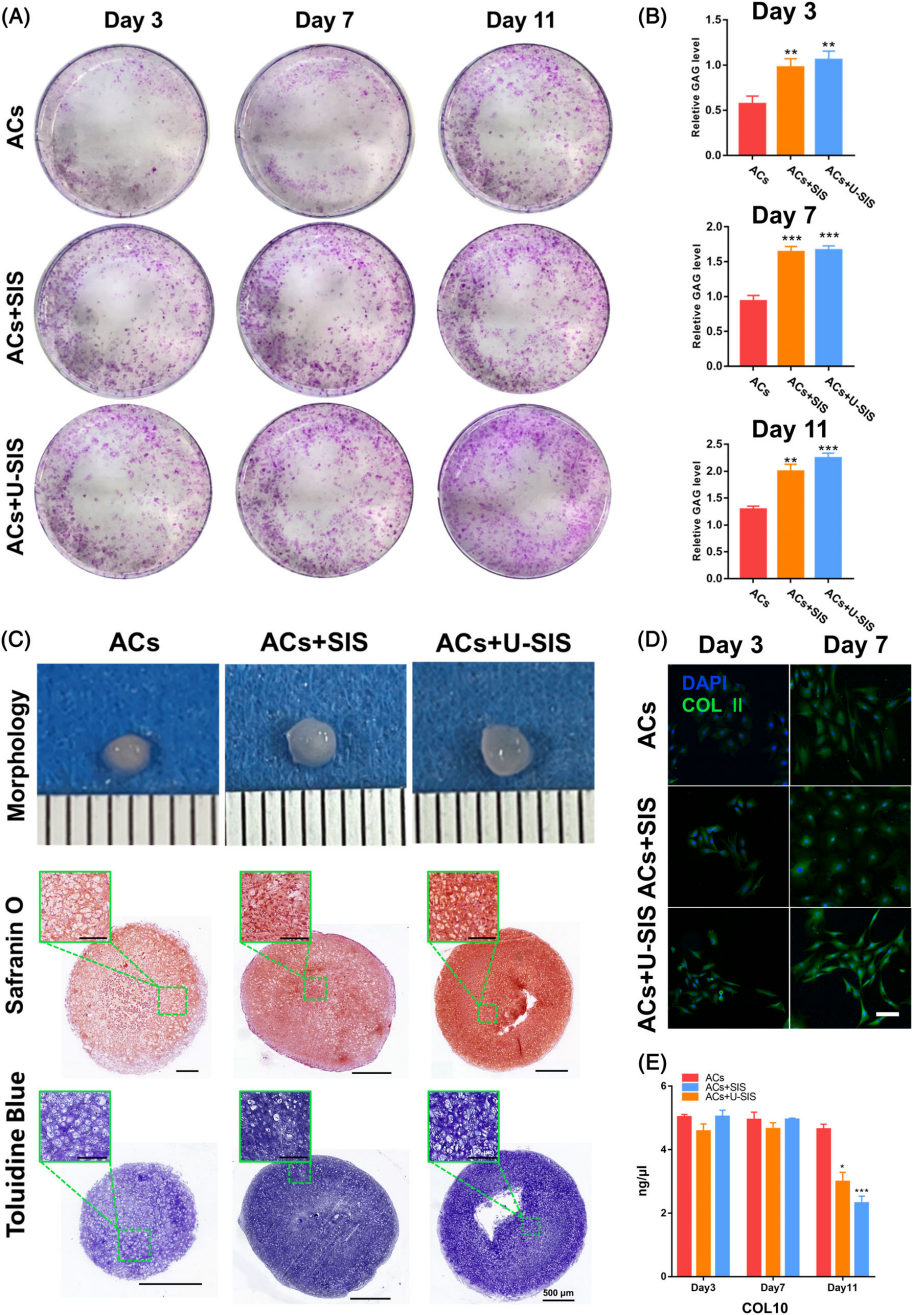
Expression of chondrogenic‐relative proteins in auricular‐derived chondrocyte cell (ACs). (A) Images and (B) Results of GAGs expression in the ACs co‐cultured with the small intestinal submucosa (SIS) and U‐SIS. *p < 0.05, **p < 0.01, ***p < 0.001. (C) Pellet culture of the ACs after 14 days of culture, including morphology, safranin O staining and toluidine blue staining. Scale bar = 500 μm. (Green square represents the high magnification area of ACs pellet, Scale bar = 100 μm). (D) Immunofluorescence staining of type II collagen in the ACs after 3 and 7 days of culture. Scale bar = 50 μm. (E) Expression of type X collagen in the ACs as detected by an ELISA kit. *p < 0.05, ***p < 0.001.

Chondrogenesis, as a vital function of the ACs, was validated with histological and molecular methods. As shown, the ACs co‐cultured with the SIS and U‐SIS in a 2D environment could secrete more GAGs and type II collagen after 3 days, and by day 11, the expression of GAGs and type II collagen in the ACs + U‐SIS group were higher than the other two groups (Figure 4A,B,D).

#### 3.2.2 Expression level of type X collagen in the ACs

As detected with an ELISA kit (Figure 4E), by day 11, the expression level of type X collagen has decreased significantly in the ACs co‐cultured with the SIS and U‐SIS. In particular, the cells in the ACs + U‐SIS group had the lowest expression of type X collagen.

#### 3.2.3 Pellet culture of the ACs

As shown in Figure 4C, the ACs have formed a pellet in cell culture medium after 14 days of culture. The pellets in all groups had a white, elastic, and ball‐like appearance. Histological examination revealed mature cartilage lacuna in the pellets of the ACs + SIS and ACs + U‐SIS groups, which were filled with GAGs. By contrast, the cartilage lacuna in the pellets of the ACs group appeared to be immature with little GAGs.

#### 3.2.4 Expression level of relative genes in the ACs

**FIGURE 5:**
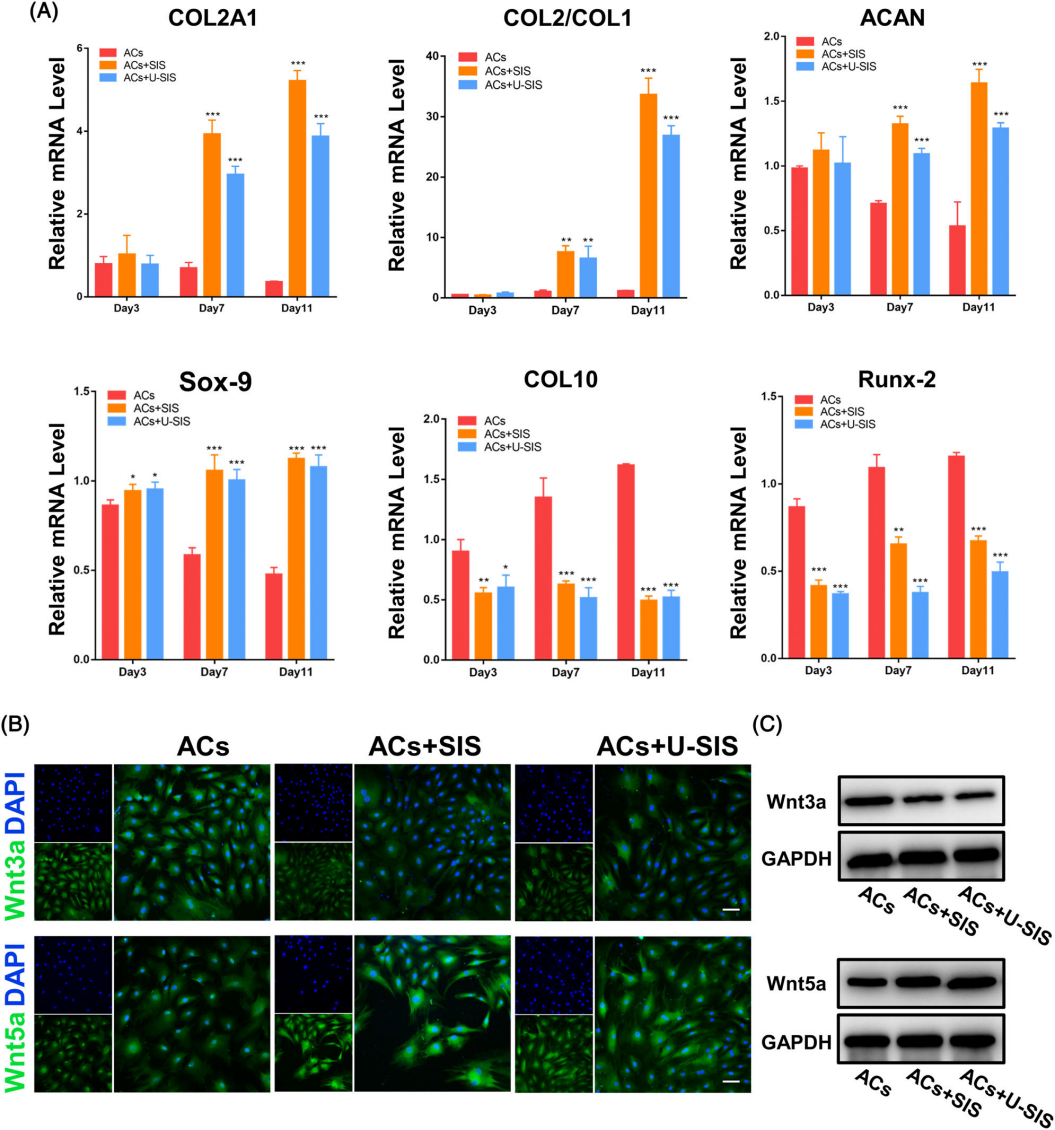
Expression of chondrogenic‐relative genes and pathway proteins in the auricular‐derived chondrocyte cell (ACs). (A) Expression of *COL2A1*, *COL2/COL1*, *ACAN*, *SOX9*, *COL10* and *RUNX2* genes in the ACs after the co‐culture. *p < 0.05, **p < 0.01, ***p < 0.001. (B) Immunofluorescence staining and (C) Western blotting for the expression level of *Wnt3a* and *Wnt5a* in the ACs after the co‐culture.

RT‐PCR was used to determine the expression level of genes associated with chondrocyte differentiation including *COL2A*, *ACAN*, *SOX9* and *COL2/COL1*. As shown in Figure 5A, on day 3, the ACs in all groups had similar expression of such genes. However, by days 7 and 11, the ACs in the ACs + SIS and ACs + U‐SIS groups had significantly higher expression of *COL2A*, *ACAN*, *SOX9* and *COL2/COL1* compared with the ACs group (*p* < 0.001).

An opposite trend was noted for *COL10* and *RUNX2* during the co‐culture (Figure 5A), which are genes associated with dedifferentiation of the ACs. The ACs showed a high expression of *COL10* and *RUNX2* genes during the culture with an increasing trend. By contrast, those of the ACs + SIS and ACs + U‐SIS groups showed a significantly lower expression of *COL10* and *RUNX2* on days 3, 7 and 11 (*p* < 0.001).

#### 3.2.5 Expression of *Wnt5a* and *Wnt3a* proteins in the ACs

By immunofluorescence staining and Western blotting, the expression of *Wnt5a* protein in the co‐cultured ACs was significantly increased in the ACs + SIS and ACs + U‐SIS groups, whilst that of *Wnt3a* protein was significantly decreased (Figure 5B,C).

### 3.3 Macroscopic appearance of the grafted diced cartilage

The SIS and U‐SIS were used to wrap the diced cartilage and graft to repair the auricular cartilage defects in the rabbit models. The animals were divided into five groups, including the control, DC, DC + P, DC + SIS and DC + U‐SIS groups (four animals each).

**FIGURE 6:**
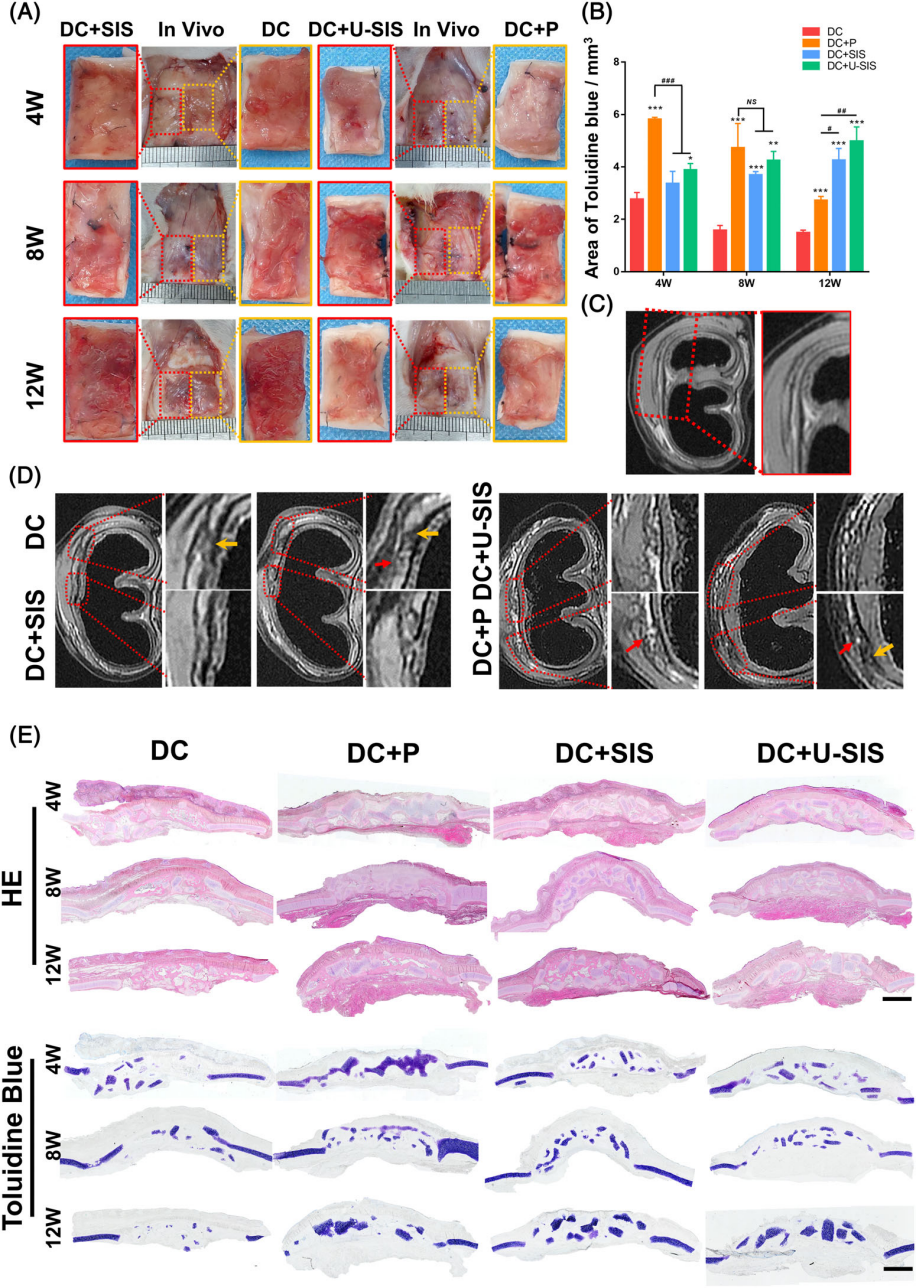
Survival and regeneration of the diced cartilage after the grafting. (A) Gross pathology of the repair area after 4, 8 and 12‐weeks. (B) Areas stained by Toluidine blue by image J. *p < 0.05, **p < 0.01, ***p < 0.001, compared with the DC group. ^#^ *p* < 0.05, ^##^ *p* < 0.01, ^###^ *p* < 0.001, compared with DC + P group. (C) MRI image of the normal auricular cartilage. (D) MRI images of repairing area at 12 weeks. Yellow arrow: Cartilage was interrupted. Red arrow: Ambiguous boundary. (E) HE staining and Toluidine blue staining of the repair area at 4, 8 and 12 weeks. Scale bar = 2 mm.

The gross pathology of the repair area was recorded after 4, 8 and 12 weeks (Figure 6A). In the DC group, the grafted diced cartilage could be seen in the repair area, but were irregular and stick out of the defects. There was also severe adhesion between the diced cartilage and surrounding tissues. After 12 weeks, no cartilage tissue could be seen in the defects, with much more stiffness compared with the surrounding cartilage tissue. In the DC + P group, the perichondrium could be seen on the top of the repairing area, and some adhesions were found between perichondrium and surrounding tissues. By the 12th week, the surface of repair area was uneven, with part of the sections being higher than the surrounding cartilage. In the DC + SIS and DC + U‐SIS groups, the repair area was flat and smooth without adhesion with surrounding tissue, with a similar height with the surrounding cartilage. At 4 weeks, the residual SIS and U‐SIS could be seen on the top of repair area, and diced cartilage could be found in the defects. At 12 weeks, both the SIS and U‐SIS have degraded completely, and the diced cartilage has completely healed by surrounding cartilage. In the control group (Figure S1), the morphology was similar to that of the DC group, but no regenerated cartilages were found in the defects by HE and Safranin O‐Fast green staining. Fibrous connective tissues have filled the defects, along with severe adherence with the surrounding tissues.

### 3.4 Survival and regeneration of the diced cartilages in vivo

To observe the regeneration of diced cartilage in vivo, MRI was applied to the animals at the 12 weeks (Figure 6D). As shown, the normal auricular cartilage was uniform in thickness, undivided and well‐defined (Figure 6C). By contrast, the cartilage of the DC group appeared to be interrupted, disorderly, and ambiguous in boundary, whilst the DC + P, DC + SIS and DC + U‐SIS groups presented better continuity. The defects in the DC + P group were ambiguous in boundary, whilst those of the DC + SIS and DC + U‐SIS groups had better‐defined boundary.

The survival and regeneration of the diced cartilages was observed by HE and Toluidine blue staining at 4, 8 and 12 weeks (Figure 6E). At 4 weeks, the diced cartilages in all groups have not filled the defects, with many connective tissues between them, which prompted absorption of part of the diced cartilage. From 4 weeks to 12 weeks, the amount of cartilage has decreased in the defects of the DC and DC + P groups. In particular, few diced cartilages in the DC group have survived to 12 weeks. In the DC + SIS and DC + U‐SIS groups, the amount of cartilage was stabilised from 4 weeks to 12 weeks. As shown by Toluidine blue staining, the amount of cartilage in the defect of the DC and DC + P groups has significantly decreased from 4 to 12 weeks, whilst that of the DC + SIS and DC + U‐SIS groups showed an increasing trend. (Figure 6B). The DC + U‐SIS group had the most amount of cartilage in the defect. The result of histological examination was consistent with that of MRI imaging.

### 3.5 Calcification of the diced cartilages in vivo

**FIGURE 7:**
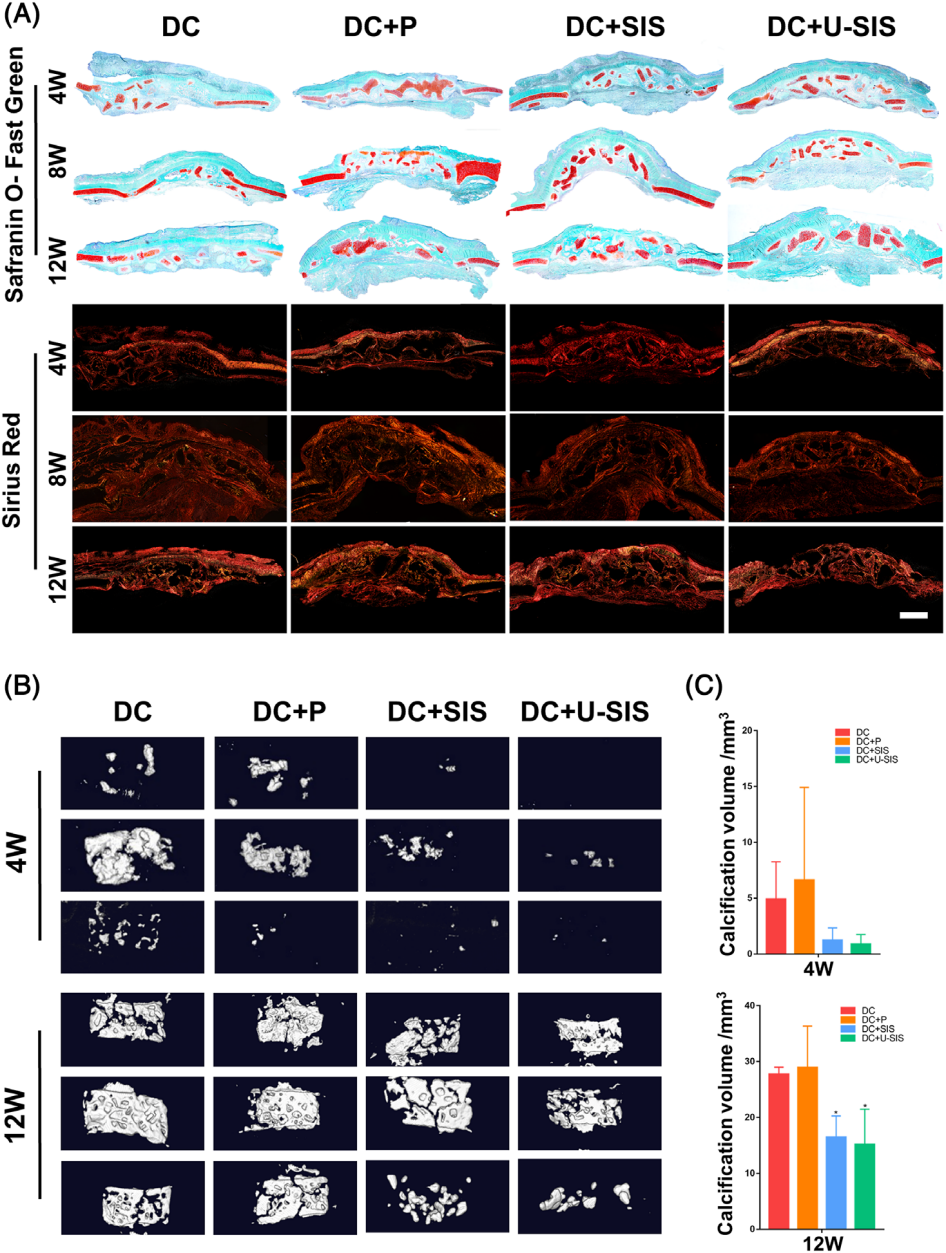
Calcification of the diced cartilage in vivo. (A) Safranin O‐Fast green and Sirius red staining of the repair area at 4, 8 and 12 weeks. Scale bar = 2 mm (B) MicroCT images and (C) calcification volume of the diced cartilage at 4 and 12 weeks. *p < 0.05.

The calcification of grafted cartilage was first detected by MicroCT at 4 and 12 weeks (Figure 7B). The grafted cartilage in all groups had calcified, with the quantity gradually increased after the operation. At 4 weeks, although no significant difference was found in the quantity of calcified tissue, the DC + SIS and DC + U‐SIS groups showed less calcified tissues. At 12 weeks, the quantity of calcified tissue in the DC + SIS and DC + U‐SIS groups became significantly less than that in the DC group, whilst no difference was found between the quantity of calcified tissues in the DC and DC + P groups (Figure 7C).

The calcification of the diced cartilages was observed by Safranin O‐Fast Green staining (Figure 7A and S2A). At 4 weeks, bone‐like tissues were found in the DC group but not in other three groups. At 8 weeks, the bone‐like tissues in the DC group have increased and matured, which were around the diced cartilage and had a morphology similar to that of bone lamella.^48^, ^49^ By contrast, there were only few bone‐like tissues beside the diced cartilage in the other three groups. At 12 weeks, the DC group showed similar calcification at 8 weeks. The bone‐like tissue in the DC + P and DC + SIS groups have increased and matured, filing the interval of the diced cartilages. The bone‐like tissues in the DC + U‐SIS group also increased, but was the least among all groups and beside the diced cartilage.

Sirius red staining was used to observe the type I collagen in the defects (Figure 7A, S2A,B). Type I collagen had shown an increasing trend in all groups from 4 to 12 week after the operation. At 4 weeks, the type I collagen fibres were thick in the DC group, particularly at the boundary of the diced cartilage. Whilst in the DC + P, DC + SIS and DC + U‐SIS groups, the fibres were thin and short and distributed in the interval of the diced cartilage. At 8 weeks, the type I collagen fibres in the DC and DC + P groups became thicker and inter‐connected, filling the space between the diced cartilages. The type I collagen fibres in the DC + SIS and DC + U‐SIS groups were significantly less compared with the DC and DC + P groups at 8 and 12 weeks.

### 3.6 Tracing of the USCs in vivo

Immunofluorescence staining of HLA‐ABC protein showed that the USCs only distributed around the defects in the DC + U‐SIS group 1 and 2 weeks after the surgery (Figure S3). However, USCs have been entirely absorbed by the fourth week. Immunofluorescent staining showed that the DC + SIS and DC + U‐SIS groups had lower expression of CD86 compared with the DC and DC + P groups 1 and 2 weeks after the surgery. And there was a higher expression of CD206 in the DC + SIS and DC + U‐SIS groups.

### 3.7 Long‐term regeneration of the diced cartilages

**FIGURE 8:**
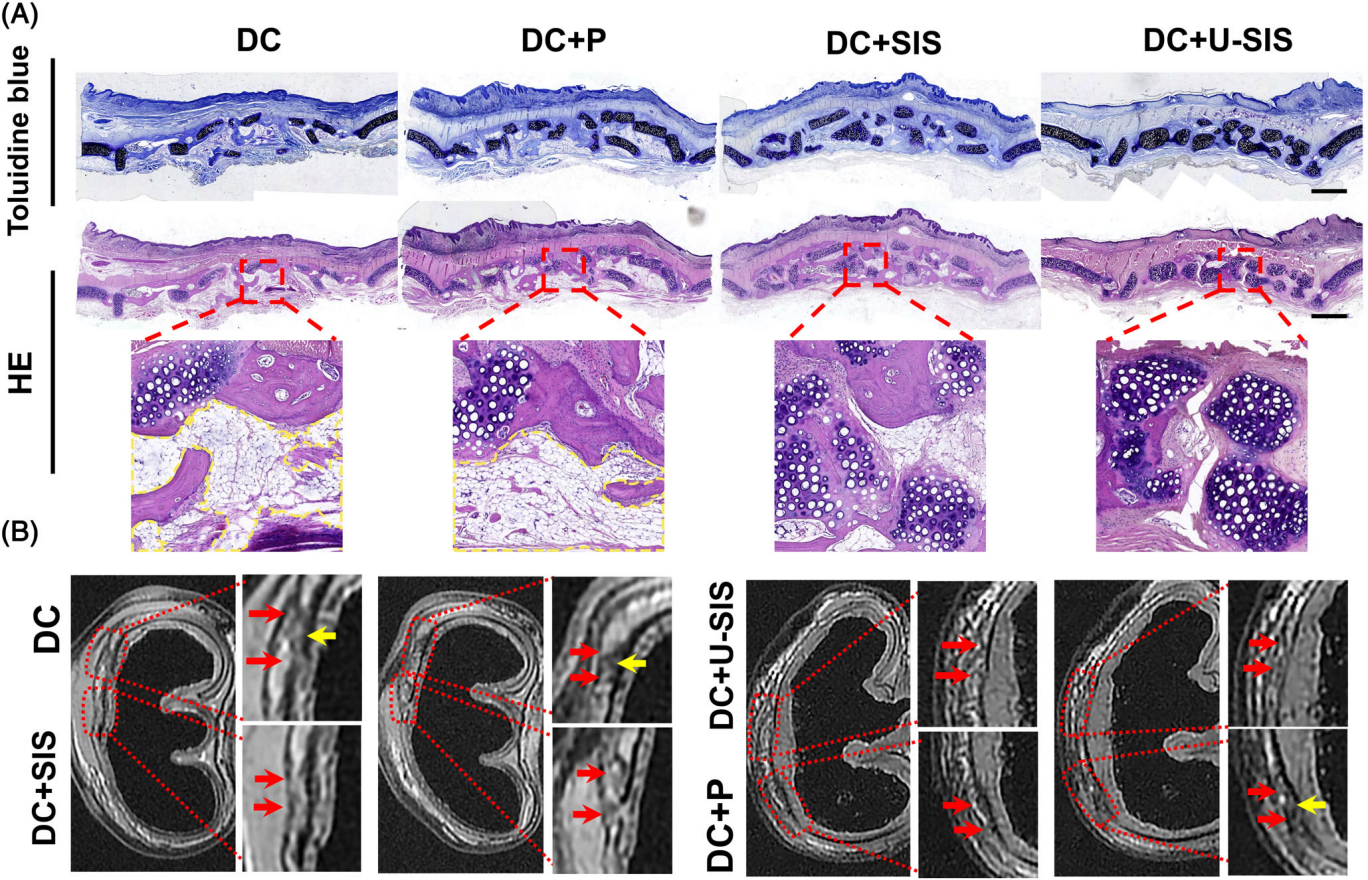
Histologic and ichnologic results of the repair area at 24 weeks. (A) Toluidine blue and HE staining of the repair area. Yellow broken line: adipose tissue in repairing area. Scale bar = 2 mm. (B) MRI images of the repair area. Yellow arrow: Cartilage was interrupted. Red arrow: Ambiguous boundary.

Twenty‐four weeks after the surgery, regeneration of the diced cartilages was noted by both MRI and histological staining (Figure 8). The amount of cartilage has increased in all groups compared with 12 weeks. The cartilage in the DC + U‐SIS group has filled the defects and shown undivided signals on MRI imaging. By contrast, the defects in the DC and DC + P groups were filled with bone‐like tissue, adipose tissue, and cartilage, with less cartilage compared with the DC + SIS and DC + U‐SIS groups, along with interrupted signals on MRI imaging.

## 4 DISCUSSION

Diced cartilage graft is a commonly used method in rhinoplasty. During the operation, there are several choices for wrapping materials, which include perichondrium,^14^ temporalis muscle fascia^50^ and rectus muscle fascia.^8^ Although many clinical trials have compared the effect of various materials in longer terms, there is still no gold standard among such materials.^51^, ^52^ These wrapping materials as autogenous tissues possess favourable biocompatibility, but with certain limitations regarding the application and effect, including the available quantity, aggravation of injury, prolonging of operating time and complications at the donor site. Therefore, we have proposed to use the SIS or U‐SIS membranes as wrapping materials for diced cartilage graft, and proved that both could promote chondrogenesis and suppress dedifferentiation of chondrocyte in vitro, increase the survival of diced cartilage in short terms, and improve its regeneration in longer terms in rabbit models.

The SIS has been widely used in recent years as a tissue engineering material, partly for it is degradable and can release various cytokines including VEGF, b‐FGF, TGF‐β and TNF‐α,^24^ which in turn can induce cascade reactions and recruit various cells from surrounding tissues to promote tissue repairing.^53^ Therefore, it has shown to promote wound healing,^54^ gastric ulcer healing,^27^ laparoscopic paraoesophageal hernia repairing^55^ and tracheal repairing.^30^ It can also promote chondrogenesis and cartilage regeneration.^30^, ^37^ Since the USCs can be derived from urine in a simple and non‐invasive way, it has a great potential to be used for clinical applications. Considering the characteristics of the USCs and chondrogenic ability of the ECM,^42^ we have attempted to enhance the chondrogenic ability of the SIS by culturing the USCs on its surface. Comparing with autogenous tissue, both SIS and USCs have an abundant resource and can simplify the operative procedure, reduce the surgical wound and shorten operative time.

Chondrocytes are highly specialised cells that can produce, sustain, and degrade cartilage ECM. In cartilage ECM, GAGs and type II collagen are the vital proteins responsible for elasticity of cartilage.^56^ When such proteins gradually loss from ECM, the chondrocytes were defined as dedifferentiation.^57^ And the dedifferentiated ACs would also have high expression of type X collagen. In vitro, we have found that the ACs showed lower concentration of GAGs and type II collagen during continuous culture with cell culture medium, whilst the secretion of GAGs and type II collagen have increased when the ACs were co‐cultured with the SIS and U‐SIS, especially in the ACs + U‐SIS group. The outcome indicated that the ACs have gradually lost the ability of secreting the GAGs and type II collagen during culture, whilst both the SIS and U‐SIS could promote ACs to secret GAGs and type II collagen. It was also observed that expression of type X collagen was stabilised at a high level in the ACs group, but has decreased significantly in the ACs + SIS (*p* < 0.05) and ACs + U‐SIS groups (*p* < 0.001). Therefore, both the SIS and U‐SIS could promote the expression of GAGs and type II collagen and suppress the expression of type X collagen in the ACs.

Based on the change of protein level, we also detected relative gene expression. In the ACs, the genes of *COL2A*, *ACAN*, *SOX9* and *COL2/COL1* are the markers of differentiation, and *COL1*, *COL10* and *RUNX2* are the markers of dedifferentiation.^58^ The freshly isolated chondrocytes usually have high expression level of *COL2A* and *ACAN* genes,^59^ which are responsible for major molecules of mature cartilage, and *SOX9* gene, which is an essential transcription factor driving differentiation and a potent inhibitor of hypertrophy.^60^ As reported by previous studies, chondrocytes have a trend for dedifferentiation during the subculturing in vitro,^60^ so that the expression level of differentiation markers will decrease whilst the dedifferentiation markers will increase,^61^ and the ratios of *COL2/COL1* will decrease.^62^ Our results revealed that the expression of *COL2A*, *ACAN*, *SOX9* and *COL2/COL1* genes were up‐regulated, and that of the *COL10*, *RUNX2* genes were down‐regulated in the ACs + SIS and ACs + U‐SIS groups. These indicated that the SIS and U‐SIS can upregulate the expression of *COL2A*, *ACAN*, *SOX9*, *COL2/COL1* genes to restore the concentrations of GAGs and type II collagen in the ECM of the ACs, and downregulate the expression of *COL10* and *RUNX2* genes to decrease the expression of type X collagen and type I collagen and prevent dedifferentiation. Moreover, the U‐SIS showed more influence on the expression of relevant genes and proteins.

The Wnt signalling pathway plays an important role in chondrocyte. Among these pathway proteins, *wnt3a* and *wnt5a* exert obvious effect on regulating chondrocyte differentiation. *Wnt3a* can cause a serious reaction in chondrocytes and lead to chondrogenic dedifferentiation including loss of *COL2A* and *ACAN* gene expression,^63^ and it can also cause significant inhabitation at the initial stages of cartilage differentiation.^64^ Therefore, *wnt3a* is an inducer of dedifferentiation in mature chondrocytes. By contrast, *wnt5a* can stimulate early stage chondrogenic differentiation and induce the expression of *SOX9*,^65^ and it can also decrease the expression of *RUNX2* to suppress chondrogenic hypertrophy.^64^, ^65^ In this study, both SIS and U‐SIS could decrease the expression of *wnt3a* and increase that of *wnt5a*, which was in consistent with the expression of protein and gene, which further confirmed that both SIS and U‐SIS can promote chondrogenic differentiation and suppress hypertrophy in vitro.

During clinical treatment, a major concern over the diced cartilage graft are irregularities of appearance and resorption of cartilage.^66^ Dorsal irregularity was a serious complication of rhinoplasty, which may result from factors including thin wrapping materials,^67^ local inflammation and a large amount of resorption.^68^ And resorption of grafted cartilage was an uncontrolled process after the operation. As the resorption rate has remained unclear, it was difficult to estimate the long‐term prognosis.^1^ Perichondrium and deep muscle fascia have been commonly used for wrapping the diced cartilage during operation, as they possess suitable thickness to cover the irregular edges of diced cartilage and have attained satisfactory outcome in long terms.^14^ In this study, we have compared the gross pathology of various materials for wrapping the diced cartilage, with the SIS and U‐SIS groups showing more regular and smoother appearance (Figure 5A) owing to the ductility, restorability, and structural feature of the SIS. And the SIS is mainly consisted of collagen fibres. When the membrane was taut, the local membrane had enough tensile strength to bound the diced cartilages. The smooth surface at the serous side of the SIS also helped to decrease the adhesion with surrounding tissues. Therefore, the SIS and U‐SIS membrane have both attained significant improvement in the appearance of diced cartilage graft.

By toluidine blue and safranin O staining, the DC + U‐SIS group showed significantly more cartilage survival among the four groups 12 weeks after operation. And the DC + SIS group also showed significantly more cartilage survival comparing with the DC and DC + P groups. All defects were filled with same amount of diced cartilage during the operation. Thus, compared with the perichondrium, the U‐SIS and SIS have attained better survival of the grafted cartilage and less cartilage resorption. On the other hand, Fast green staining and MicroCT both indicated that the diced cartilage has started to calcify after grafting (Figure 6B), which was in keeping with previous observation.^50^ Calcification of the graft cartilage was also a factor leading to cartilage resorption.^50^ In the DC + SIS and DC + U‐SIS groups, the calcification was not obvious at 4 and 8 weeks, suggesting that the SIS and U‐SIS membrane could delay the calcifying process after the grafting. And the extent of calcification in the two groups was significantly less compared with the DC and DC + P groups at 12 weeks, which further proved that the SIS and U‐SIS could suppress the calcification of grafted diced cartilage.

In this study, we have extended our observation to 24 weeks in vivo in order to observe the long‐term effects of the SIS and U‐SIS membranes, which was less reported in previous studies. Comparing with 12 weeks, the amount of cartilage tissue and volume of diced cartilage has increased in the four groups at 24 weeks, suggesting that cartilage tissue had regenerated during the period of 12 weeks to 24 weeks. The DC + U‐SIS group showed the most cartilage tissues in the defects, and the DC + SIS group showed more cartilage compared with the DC and DC + P groups. Moreover, probably because few adipose tissues have adhered to perichondrium, there were many adipose tissues in the defects of the DC + P group, suggesting that wrapping materials from autologous tissue are heterogeneous in origin and may bring other tissues into the cartilage and suppress the regeneration. By contrast, as both SIS and U‐SIS membranes were simple tissues, which reduced their possibility to carry other tissues and provided a separate space for cartilage regeneration.

In summary, we have verified that both SIS and U‐SIS can promote chondrocyte differentiation and inhibit chondrocyte dedifferentiation in vitro, thereby confirmed their feasibility for wrapping the diced cartilage and grafting in rabbit models. In animal experiments, the histologic and imaging results have proved that the SIS and U‐SIS as wrapping materials could improve the survival rate of diced cartilage, reduce cartilage resorption and inhibit cartilage calcification shortly after the grafting of the diced cartilage, and promote cartilage regeneration in longer terms. Moreover, both the SIS and U‐SIS membranes had lower chance to bring other tissues into the grafted cartilage. Based on these, we conclude that the SIS and U‐SIS materials have the potential to be used for wrapping the diced cartilage and grafting, though this effect still requires longer term observation in larger animals.

## AUTHOR CONTRIBUTIONS

Shang Li contributed to conceptualisation, methodology, investigation, software, validation, data curation, formal analysis, writing–original draft, and visualisation. Rui Wang involved in conceptualisation, methodology, resources, validation, formal analysis, writing–review and editing. Liping Huang and Yanlin Jiang performed methodology. Fei Xing performed data curation, formal analysis, writing–review and editing. Weiqiang Duan contributed to conceptualisation and methodology. Ying Cen contributed to conceptualisation, writing–review and editing. Zhenyu Zhang performed conceptualisation and methodology. Huiqi Xie involved in conceptualisation, writing–review and editing, supervision, project administration, and funding acquisition. All authors read and approved the final manuscript.

## FUNDING INFORMATION

This work was supported by the National Natural Science Foundation of China (32171351), the “1.3.5” Project for Disciplines of Excellence, West China Hospital, Sichuan University (ZYJC18002, ZYPY20003 and ZYPY20004), Natural Science Foundation of Sichuan Province (2022NSFSC0717), Sichuan Science and Technology Program (2023YFS0063), the Frontiers Medical Center, Tianfu Jincheng Laboratory Foundation (TFJC2023010002), and National Natural Science Foundation of China (82202705).

## CONFLICT OF INTEREST STATEMENT

The authors declare that they have no known competing financial interests or personal relationships that could have appeared to influence the work reported in this article.

## Supporting information


**Data S1.** Supporting Information.

## Data Availability

All data generated or analyzed during this study are included in this published article and its supplementary information files.
