# Supplementary material for: Promotion of diced cartilage survival and regeneration with grafting of small intestinal submucosa loaded with urine‐derived stem cells
Source: Cell Prolif. 2023 Sep 18;57(2):e13542. doi: 10.1111/cpr.13542 (PMC10849789; doi:10.1111/cpr.13542)
Supplement: Supplementary file 1 — Data S1. Supporting Information. [file CPR-57-e13542-s001.docx]

**Supplementary Material**

**Promotion of Diced Cartilage Survival and Regeneration with Grafting of Small Intestinal Submucosa Loaded with Urine-Derived Stem Cells**

Shang Li^†^, Rui Wang^†^, Liping Huang, Yanlin Jiang, Fei Xing, Weiqiang Duan, Ying Cen, Zhenyu Zhang^*^, Huiqi Xie^*^


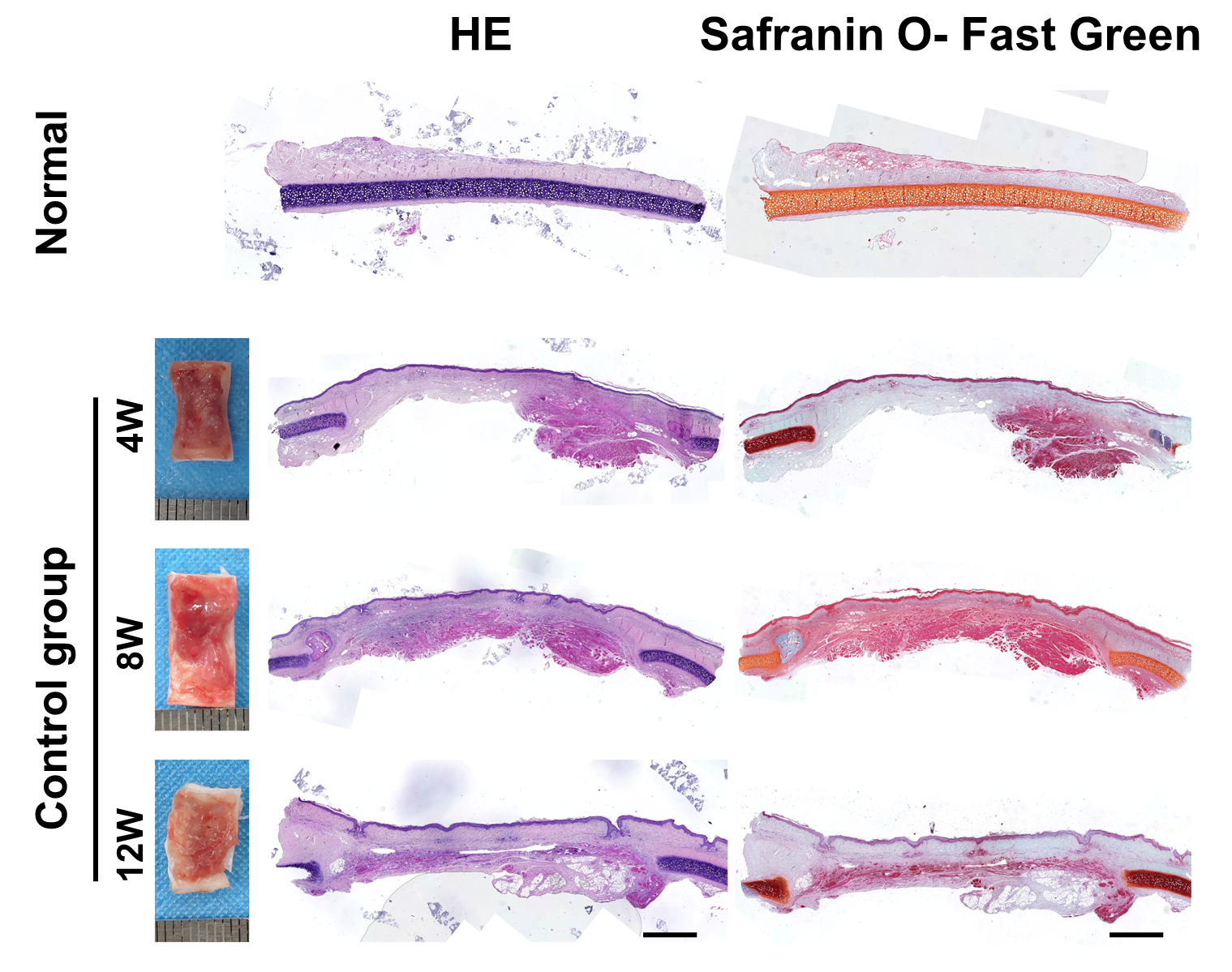


**Supplementary Figure 1. Morphology and histology of the normal cartilage and control groups.** Scale bar = 2 mm


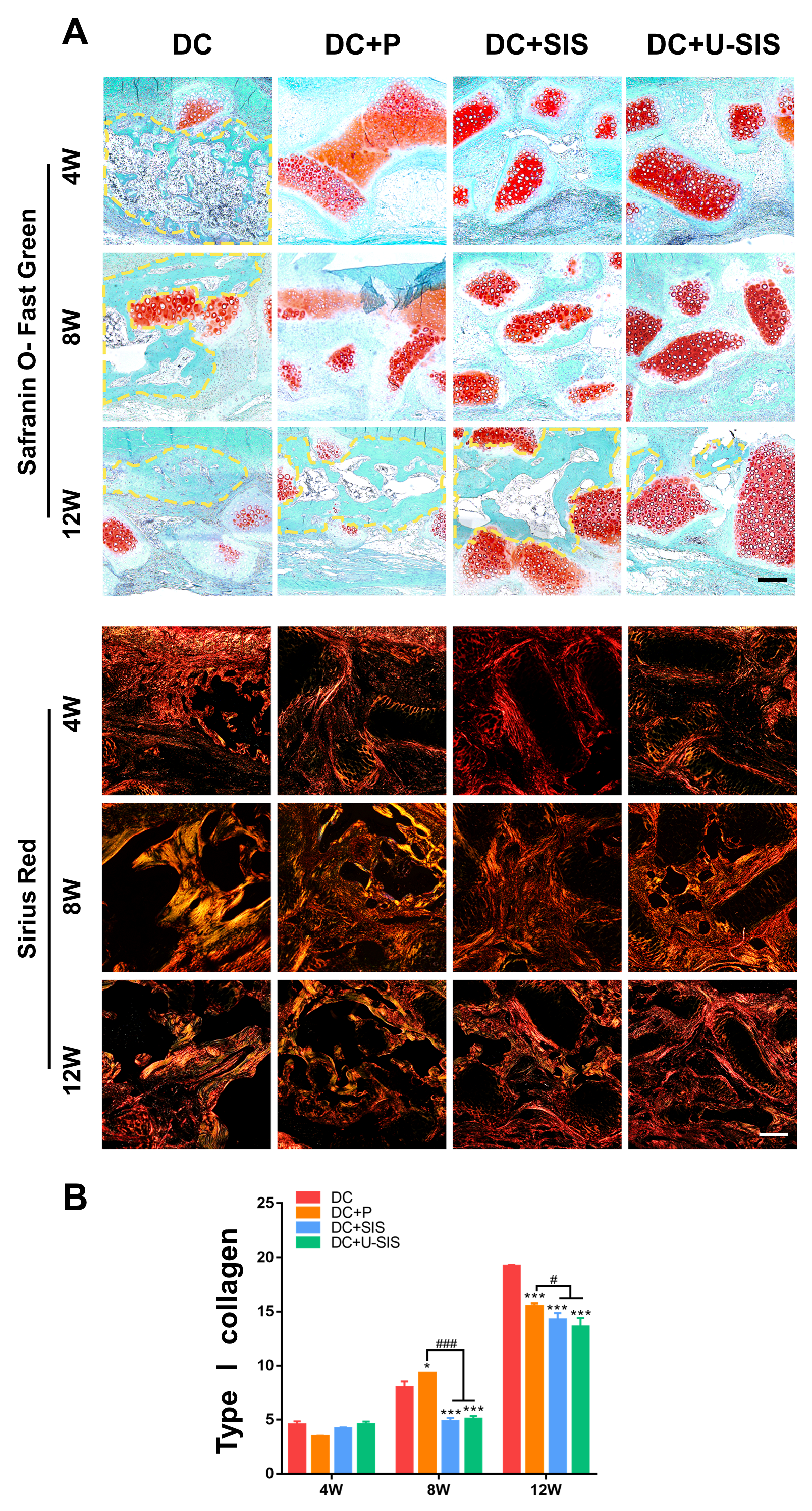


**Supplementary Figure 2. Calcification of the diced cartilage *in vivo*.** (A) Safranin O-Fast green and Sirius red staining of the repair area at 4, 8, and 12 weeks. Scale bar = 500 μm. Yellow broken line: bone-like tissue in repairing area. (B) Type Ⅰ collagen in the repair area with Sirius red staining. ^***^*P* < 0.001, compared with the DC group. ^#^*P* < 0.05, ^###^*P* < 0.001, compared with the DC+P group


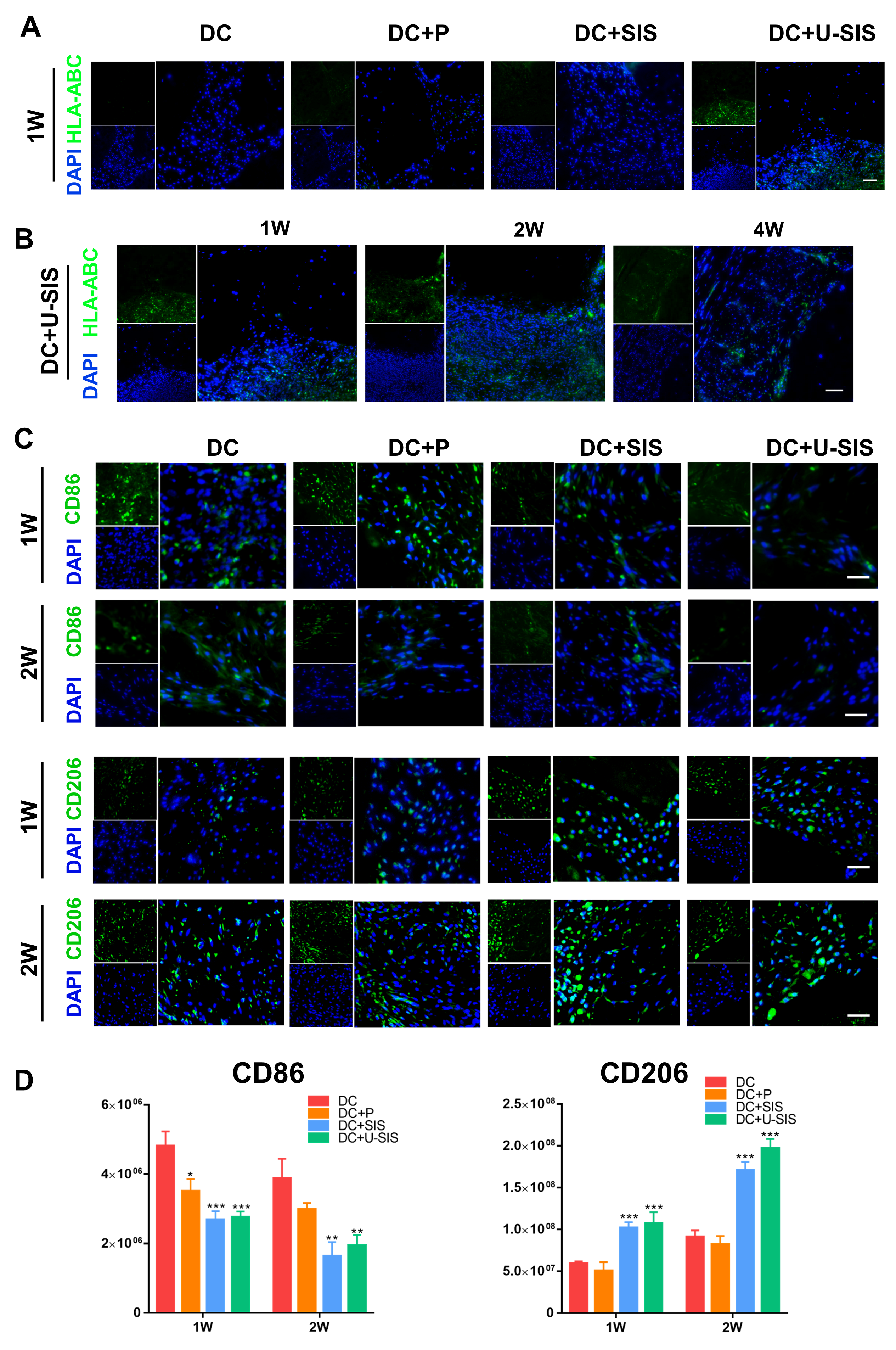


**Supplementary Figure 3. Tracing of the USCs and local inflammation**. (A) Immunofluorescence results of HLA-BCA of the DC, DC+P, DC+SIS, and DC+U-SIS groups after 1 week. Scale bar = 50 μm. (B) Immunofluorescence results of the HLA-BCA of the DC+U-SIS group after 1, 2, and 4 weeks. Scale bar = 50 μm. (C) Immunofluorescence results and (D) statistic results of CD86 and CD206 after 1 and 2 weeks. Scale bar = 50 μm. ^*^*P* < 0.05, ^**^*P* < 0.01, ^***^*P* < 0.001

**Table S1. PCR primers of the selected genes**

| Gene | Primer sequence (5’→3’) |
| --- | --- |
| *GAPDH* | F: CCATTCATTGACCTCCACTACATG |
|  | R: CGTACTGGGCACCAGCATC |
| *COL1A1* | F: ATGGTGCCAAGGGAGATGC |
|  | R: TTAGCACCGACAGCTCCAGG |
| *COL2A1* | F: CAACAGCAGGTTCACCTATACCG |
|  | R: ACCGGTACTCGATGACAGTCTTG |
| *SOX9* | F: AATCTCCTGGACCCCTTCAT |
|  | R: GTCCTCCTCGCTCTCCTTCT |
| *ACAN* | F: AGAGGCCGAGTGGATCCAG |
|  | R: TGTGACGCGTCCCTCTGTC |
| *COL10* | F: GTTCATGGAGTGTTCTACGCTGAG |
|  | R: ACCTTGTTCTCCTCTCACTGG |
| *RUNX2* | F: GACTGTGGTTACCGTCATGGC |
|  | R: ACTTGGTTTTTCATAACAGCGGA |
